# Evaluating the Contribution of Cell Type–Specific Alternative Splicing to Variation in Lipid Levels

**DOI:** 10.1161/CIRCGEN.120.003249

**Published:** 2023-05-11

**Authors:** Katerina A.B. Gawronski, William P. Bone, YoSon Park, Evanthia E. Pashos, Brandon M. Wenz, Max F. Dudek, Xiao Wang, Wenli Yang, Daniel J. Rader, Kiran Musunuru, Benjamin F. Voight, Christopher D. Brown

**Affiliations:** Cell and Molecular Biology Graduate Group (K.A.B.G., B.M.W.), University of Pennsylvania - Perelman School of Medicine, Philadelphia, PA; Genomics and Computational Biology Graduate Group (W.P.B., M.F.D.), University of Pennsylvania - Perelman School of Medicine, Philadelphia, PA; Department of Genetics (Y.P., E.E.P., D.J.R., K.M., B.F.V., C.D.B.), University of Pennsylvania - Perelman School of Medicine, Philadelphia, PA; Cardiovascular Institute (X.W.), University of Pennsylvania - Perelman School of Medicine, Philadelphia, PA; Institute for Regenerative Medicine (W.Y.), University of Pennsylvania - Perelman School of Medicine, Philadelphia, PA; Department of Medicine (D.J.R., K.M.), University of Pennsylvania - Perelman School of Medicine, Philadelphia, PA; Division of Translational Medicine & Human Genetics (D.J.R.), University of Pennsylvania - Perelman School of Medicine, Philadelphia, PA; Department of Systems Pharmacology and Translational Therapeutics (B.F.V.), University of Pennsylvania - Perelman School of Medicine, Philadelphia, PA; Institute for Translational Medicine and Therapeutics (B.F.V.), University of Pennsylvania - Perelman School of Medicine, Philadelphia, PA

**Keywords:** alternative splicing, hepatocyte, induced pluripotent stem cells, lipids, lipoproteins

## Abstract

Genome-wide association studies have identified hundreds of loci associated with lipid levels. However, the genetic mechanisms underlying most of these loci are not well-understood. Recent work indicates that changes in the abundance of alternatively spliced transcripts contribute to complex trait variation. Consequently, identifying genetic loci that associate with alternative splicing in disease-relevant cell types and determining the degree to which these loci are informative for lipid biology is of broad interest.

**Methods:**

We analyze gene splicing in 83 sample-matched induced pluripotent stem cell (iPSC) and hepatocyte-like cell lines (n=166), as well as in an independent collection of primary liver tissues (n=96) to perform discovery of splicing quantitative trait loci (sQTLs).

**Results:**

We observe that transcript splicing is highly cell type specific, and the genes that are differentially spliced between iPSCs and hepatocyte-like cells are enriched for metabolism pathway annotations. We identify 1384 hepatocyte-like cell sQTLs and 1455 iPSC sQTLs at a false discovery rate of <5% and find that sQTLs are often shared across cell types. To evaluate the contribution of sQTLs to variation in lipid levels, we conduct colocalization analysis using lipid genome-wide association data. We identify 19 lipid-associated loci that colocalize either with an hepatocyte-like cell expression quantitative trait locus or sQTL. Only 2 loci colocalize with both a sQTL and expression quantitative trait locus, indicating that sQTLs contribute information about genome-wide association studies loci that cannot be obtained by analysis of steady-state gene expression alone.

**Conclusions:**

These results provide an important foundation for future efforts that use iPSC and iPSC-derived cells to evaluate genetic mechanisms influencing both cardiovascular disease risk and complex traits in general.

Genome-wide association studies have identified hundreds of loci associated with plasma lipid levels, an important set of predictive and causal risk factors for cardiovascular disease.^1^ The majority of variants associated with these traits are found in the noncoding genome and are hypothesized to mechanistically influence complex traits through changes in gene expression. For example, at *SORT1,* an established locus associated with both LDL (low-density lipoprotein) cholesterol levels and coronary heart disease, a functional noncoding variant creates a novel CCAAT/enhancer binding protein (C/EBP) binding site, leading to changes in hepatic *SORT1* expression and in turn, changes in circulating LDL cholesterol levels.^2^ Motivated by this and other examples, significant effort has been dedicated to identifying variants associated with changes in gene expression (expression quantitative trait loci; eQTLs) that are also associated with changes in plasma lipid levels.

Recent research indicates that variants associated with changes in the proportion of alternatively spliced transcript isoforms, or splicing quantitative trait loci (sQTLs) can provide another contributing mechanism underlying complex traits and may be as informative as total gene expression quantitative trait loci in some cases.^3,4^ For example, sQTLs discovered in lymphoblast cell lines are enriched in multiple sclerosis genome-wide association studies (GWAS) disease loci, and other studies report similar findings for sQTL enrichment in GWAS for schizophrenia and type-2 diabetes.^4–6^ Variants affecting splicing have also been linked to systemic lupus erythematosus and fatty acid metabolism.^7–9^ Splicing is an attractive mechanism for study because it can be targeted with antisense oligonucleotides, several of which are currently undergoing clinical trials to treat diseases resulting from aberrant splicing such as Duchenne Muscular Dystrophy and Spinal Muscular Atrophy.^10^ Finally, splicing provides an even finer level of detail on biological mechanism that cannot always be inferred through bulk expression analysis alone. For example, while the Alzheimer disease-associated gene *ABCA7* has both an eQTL and sQTL association, the sQTL is hypothesized to be the causal variant associated with a nonfunctional transcript.^11,12^

Functional studies of QTLs have been hindered by the time and energy required to identify and then mechanistically characterize the QTLs in model systems. QTL discovery efforts in primary tissues have been highly productive but have several important drawbacks, such as the reliance on heterogeneous postmortem tissue collection and the difficulty of interrogating phenotypes in tissues. However, it is now possible to generate individual-specific, renewable, induced pluripotent stem cell (iPSC) lines, which can be used to both identify and characterize QTLs in specific cell types. iPSCs can be differentiated into a variety of cell types, including hepatocyte-like cells (HLCs).^13^ Given the liver’s importance in the synthesis and uptake of lipids,^14^ using HLCs as a model to understand the mechanisms underlying genetic associations with lipid levels is of particular interest. Previous research has demonstrated the utility of these cell models by identifying and characterizing eQTLs in these HLCs.^15,16^

Therefore, this report has two goals, namely (1) to evaluate the feasibility of using HLC/iPSC lines for sQTL discovery, and (2) to determine whether the sQTLs discovered are informative for our understanding of lipid biology. Therefore, we mapped sQTLs in 83 individual-matched iPSC lines and iPSC-derived HLCs, on which we previously conducted genotyping and paired-end RNA-sequencing. To evaluate the degree to which the genetic control of splicing information compares to total gene expression, we also perform a variety of analyses evaluating differential splicing, differential expression, and colocalization analysis with both HLC sQTLs and HLC eQTLs. We then highlight two loci where the colocalization of GWAS and sQTL results inform the mechanism underlying the variant-trait association.

## Methods

The data that support the findings of this study are available from the corresponding author upon reasonable request. Summary results generated for this report are anonymized and deidentified and are available at: doi.org/10.5281/zenodo.7864256. Data utilized in this study were derived from a previous report.^15^ Briefly, the University of Pennsylvania Human Subjects Research Institutional Review Board approved the previous study protocol used to generate the lines, and all subjects gave written informed consent for study participation. Full methods for the presented are provided in Supplemental Material.

## Results

### iPSC and HLC Samples Have Distinct Profiles of Gene Expression and Alternative Splicing

**Figure 1.**
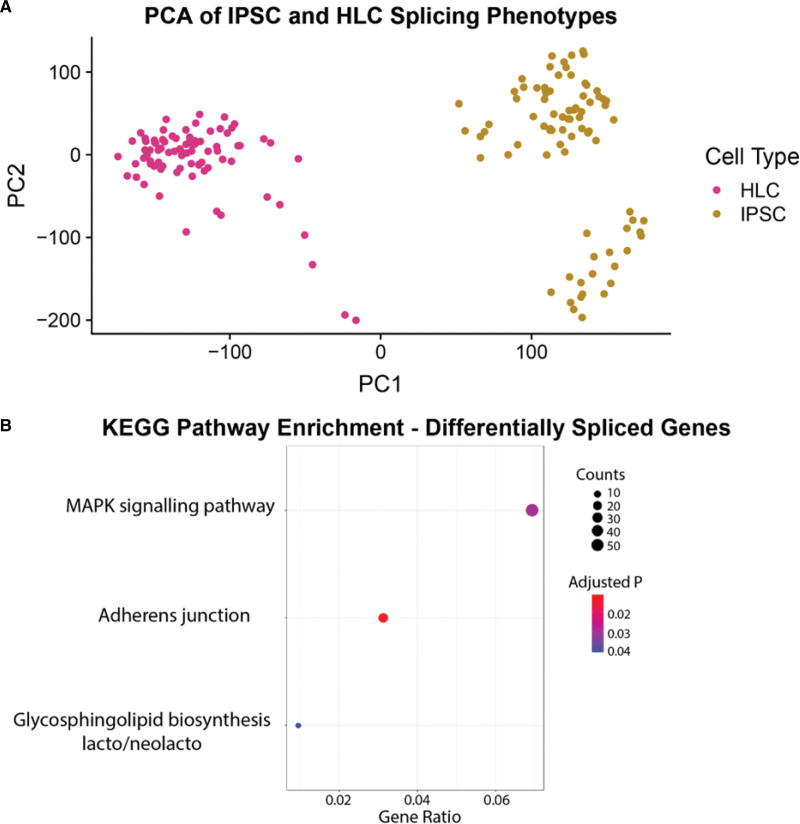
**Properties of cell lines across alternative splicing.** Differential splicing analysis: (**A**) Principal component analysis (PCA) of normalized splicing phenotypes; (**B**) enrichment of Kyoto Encyclopedia of Genes and Genomes (KEGG) pathways for differentially spliced genes with an absolute value of delta percent spliced in≥0.1 and significant at false discovery rate 5%. *P* values were adjusted for multiple testing using the Benjamini and Hochberg correction. HLC indicates hepatocyte-like cell; iPSC, induced pluripotent stem cell; and MAPK, mitogen-activated protein kinase.

We first sought to compare the patterns of alternative splicing and gene expression between iPSCs and HLCs. After normalization and standardization procedures (Supplemental Material), we applied Leafcutter to identify genes with differences in proportions of alternatively spliced transcripts between cell types. Five thousand nine hundred eighty-six genes out of 11 732 genes tested are differentially spliced between iPSC and HLC samples (false discovery rate, FDR <5%). The first two principal components of the quantile normalized and standardized splicing proportions clearly separate the samples by cell type (Figure 1A). Gene ontology analysis also demonstrated that differentially spliced genes that had at least 10% splicing difference across cell types were enriched for metabolism-relevant Kyoto Encyclopedia of Genes and Genomes (KEGG) pathways such as glycosphingolipid biosynthesis (Figure 1B).

We next compared patterns of gene expression between the iPSC and HLC lines (Supplemental Material). Virtually all (22 134 out of 23 857) tested genes were differentially expressed between iPSCs and HLCs (FDR<5%), with 6168 having an absolute log_2_ fold change of ≥2. Differentially expressed genes that were more highly expressed in HLCs (relative to iPSCs) were enriched for KEGG pathways pertaining to hepatocyte function such as cholesterol and drug metabolism (Figure S1C). The first two principal components obtained from the normalized expression profiles for the iPSCs and HLCs clearly separated samples by cell type (Figure S1A). In sum, we found substantial differences in alternative splicing and gene expression between iPSC and HLCs, and the top differentially expressed or alternatively spliced genes were enriched for liver, lipid, and metabolism-relevant pathways. These results indicate that the successful differentiation of the iPSCs into HLCs results in broad changes in steady-state transcriptional and post-transcriptional regulation.

### Thousands of sQTLs Identified Across iPSC and HLC Samples

To identify genetic variants associated with differences in the proportion of alternatively spliced transcripts (ie, sQTLs), we conducted sQTL discovery scans in both cell types using QTLtools’ permutation-based scan to identify the most highly associated differentially spliced intron per gene (Supplemental Material). After filtering for intron clusters that map to annotated genes, we obtained 13 077 genes with testable introns in HLCs and 12 543 genes with testable introns in iPSCs. We identify 1384 sQTLs in HLCs and 1455 sQTLs in iPSCs (FDR<5%). iPSC sQTLs correspond to 1436 unique sentinel single nucleotide polymorphisms (SNPs) and 1455 unique genes, whereas HLC sQTLs correspond to 1368 unique sentinel SNPs and 1384 unique genes.

To evaluate the extent to which iPSC and HLC sQTLs are discoverable in primary liver tissue, we performed replication analysis in the Gene-Tissue Expression project v6 primary liver samples (n=96).^17^ Compared to iPSC sQTLs, HLC sQTLs were slightly more likely to be replicated at the gene level in primary liver tissue as expected (π_1_=0.78 for HLC sQTLs versus π_1_=0.66 for iPSC sQTLs). These results demonstrate that sQTL discovery in iPSC and HLC samples is feasible and that both iPSC and HLC sQTLs replicate in primary liver tissue, with HLC sQTLs replicating at a higher degree than iPSCs.

### sQTLs Are Found Near Their Associated Splice Event and Are Enriched for Splicing Relevant Annotations

**Figure 2.**
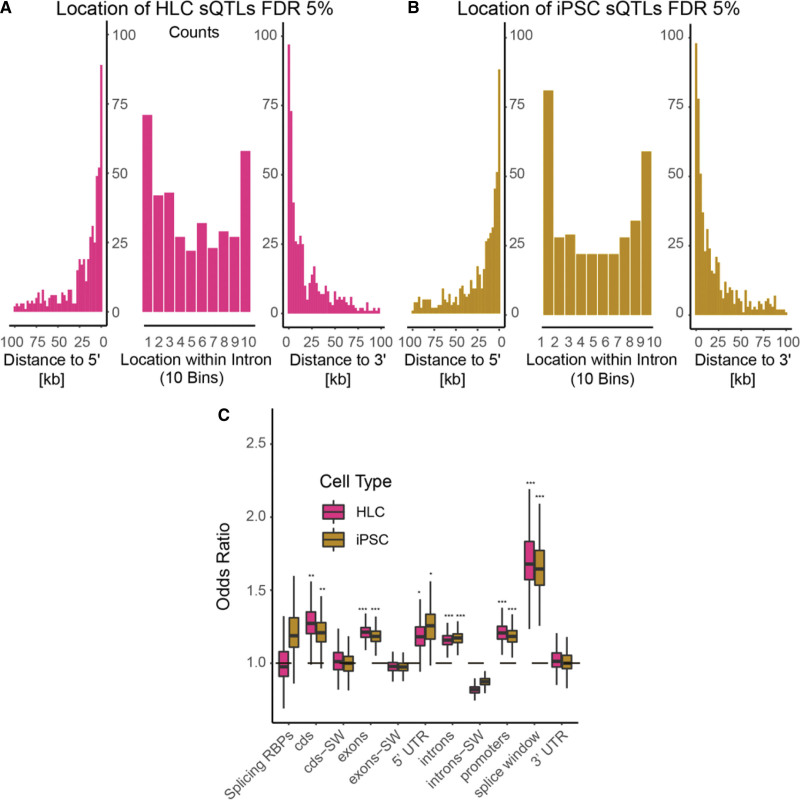
**Genomic characteristics of splicing quantitative trait loci (sQTL) discoveries in surveyed cell model systems.** Characteristics of false discovery rate 5% splicing quantitative trait loci (sQTLs): (**A**) distance of induced pluripotent stem cell (iPSC) sQTLs to splice event, (**B**) distance of hepatocyte-like cell (HLC) sQTLs to splice event, (**C**) enrichment of iPSC/HLC sQTLs in genomic annotations; boxplot whiskers calculated as interquartile range×1.5. ***P<0.001; **P<0.01; *P<0.05; annotations with “-SW” indicate that the splice window annotation track has been subtracted. RBPs indicate RNA-binding proteins; and UTR, untranslated region.

Because alternative splicing involves protein regulatory machinery interacting with genomic elements near intron/exon boundaries, we expect true sQTL variants to be found predominantly in the regions near these boundaries. Thus, we assessed the genomic context of each sQTL variant and measured the distance between each variant and its associated splice event. The majority of sentinel sQTL variants (75%) are within 25 kb of their associated splice event, although the sentinel variant may not be the ultimate causal variant. We observe similar patterns for sQTLs that fall within the intronic region of their splice event (in between the two exons that delineate the excised intron). After binning to normalize for intron length, we observe that the first and 10th deciles that are closest to canonical splice sites contain the highest numbers of sQTLs (Figure 2A and 2B). Finally, both sets of HLC and iPSC sQTLs are significantly enriched in exonic/intronic regions encompassing canonical splice sites and in several other genomic annotations (Figure 2C). These observations demonstrate that our identified sQTLs are often found close enough to canonical splicing regulatory elements to facilitate fine-mapping of causal variants and genes.

We also conducted credible set fine-mapping analysis of the colocalized sQTLs to identify 95% credible set SNPs; 75% of the colocalized sQTLs have a credible set SNP located within the sQTL-associated gene. Median credible set sizes for iPSC and HLC sQTLs were comparable to that of iPSC and HLC eQTLs—median credible set size for sQTLs is 10 and 9 SNPs for iPSC and HLC samples, respectively; median credible set size for eQTLs is 12 and 10 SNPs for iPSC and HLC samples. This suggests that sQTLs may be no more difficult to fine-map and functionally characterize than eQTLs.

### Majority of sQTLs Are Shared Between iPSCs and HLCs

**Figure 3.**
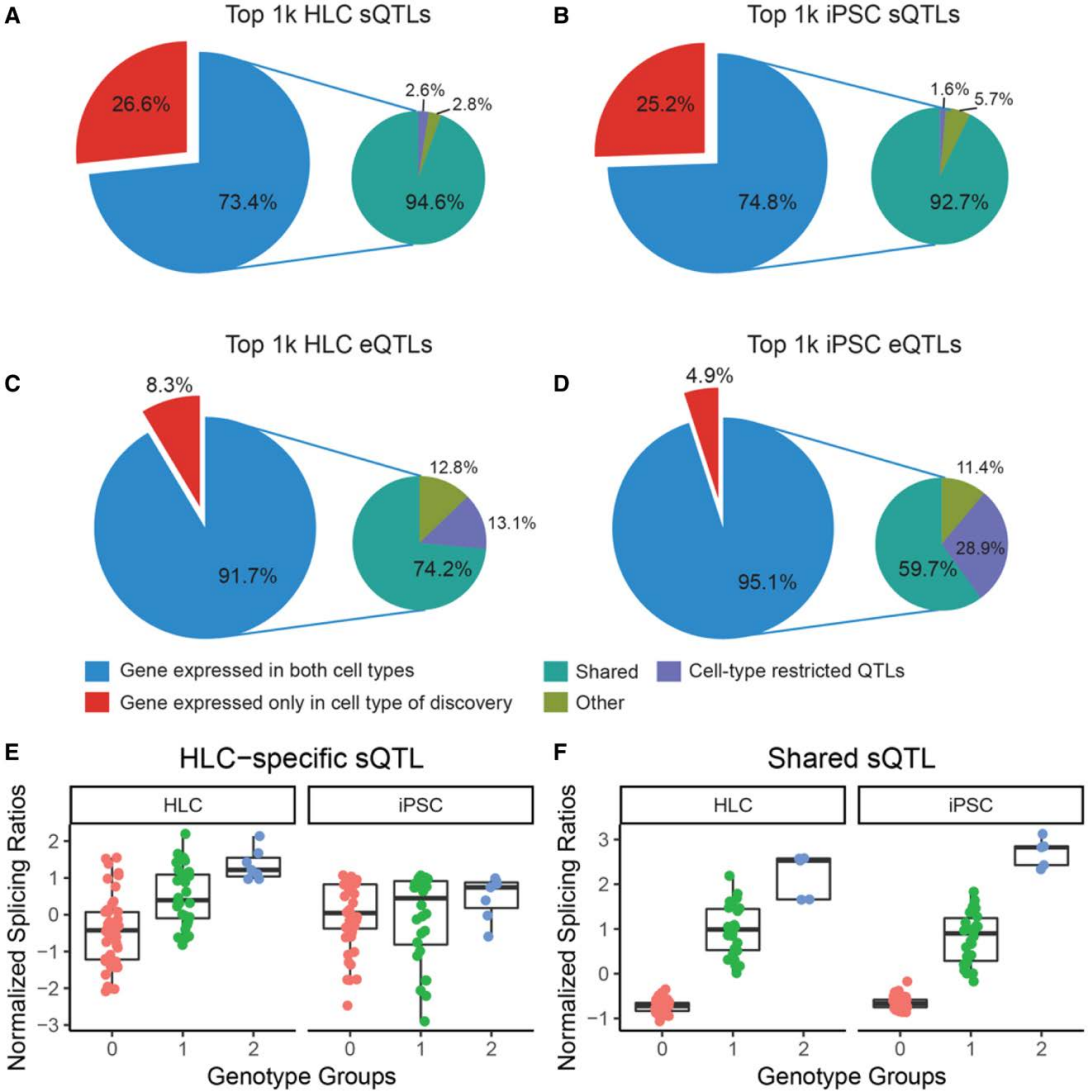
**Sharing of splicing quantitative trait loci (sQTL) and expression quantitative trait loci (eQTL) discoveries in surveyed cell model systems.** Percentage of the top 1000: (**A**) hepatocyte-like cell (HLC) sQTLs, (**B**) induced pluripotent stem cell (iPSC) sQTLs (**C**) HLC eQTLs, (**D**) iPSC eQTLs, (**E**) sQTL that is present in HLCs only, (**F**) sQTL that is identified in both HLCs and iPSCs.

Previous research has indicated that sQTL effects are often shared across tissues.^18,19^ To evaluate the extent of sQTL sharing in our cell types, we examined the top one thousand sQTLs from the FDR<5% sQTL set for both the iPSC and HLC samples and used METASOFT to determine the extent to which sQTLs discovered in one cell type are found in the other. We find that if an alternatively spliced isoform is expressed in both cell types, the sQTL effect is observed in both cell types more than 90% of the time (Figure 3A and 3B). In contrast, 60% to 74% of eQTL effects are shared, appearing to be more cell type restricted (Figure 3C and 3D). However, this difference may simply be due to statistical power for discovery. The power to detect sQTLs is lower than that of eQTLs, due to a higher burden of multiple testing correction (Supplemental Material). In the Gene-Tissue Expression project, tissues with larger sample sizes report higher percentages of tissue-specific eQTLs compared to tissues with smaller sample sizes, indicating that power is correlated with tissue-specific eQTL discovery. The high percentage of shared sQTLs identified in this study may indicate similar power limitations.

### HLC sQTLs Identify Trait-Relevant Genes and Mechanisms at GWAS Lipid Loci

To determine if our discovered sQTLs map to previously established associations for lipid traits in humans, we evaluated the colocalization probability that sQTL and GWAS signals share underlying causal architecture using coloc R package.^20^ Colocalization analysis of HLC sQTLs (FDR<5%) with genome-wide significant (*P*<5×10^-8^) loci from the Global Lipids Genetics Consortium identified 5, 3, 4, and 5 HLC sQTL genes colocalizing with HDL (high-density lipoprotein), LDL, triglycerides, and total cholesterol loci respectively (using a posterior probability [PP] cutoff of PP4/PP3+PP4≥0.9 and PP3+PP4≥0.8, Table 1).^1^ Colocalization analysis of FDR <5% HLC eQTLs identified 3, 5, 2, and 5 eQTL genes colocalized loci for HDL, LDL, triglycerides, and total cholesterol respectively (Table 2). Encouragingly, we observed colocalization of sQTL and eQTLs in genes with known roles in lipid biology (*APOC1, ANGPTL3).*^15,21^ In general, the colocalized sQTLs and eQTLs are associated with different genes, a result that is in line with previous studies.^19^ Only 2 genes colocalized for both an eQTL and sQTL, *RPAP2* and *DR1*.

**Table 1.**
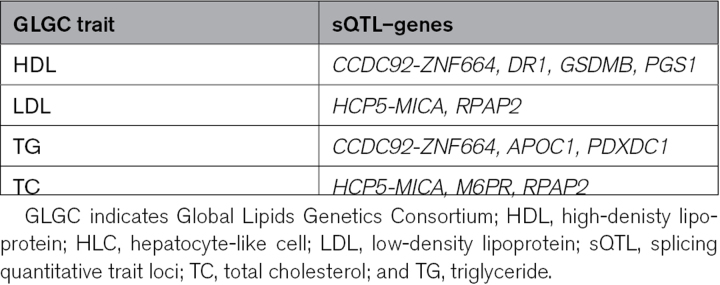
HLC sQTL Genes That Colocalize With GLGC Loci

**Table 2.**
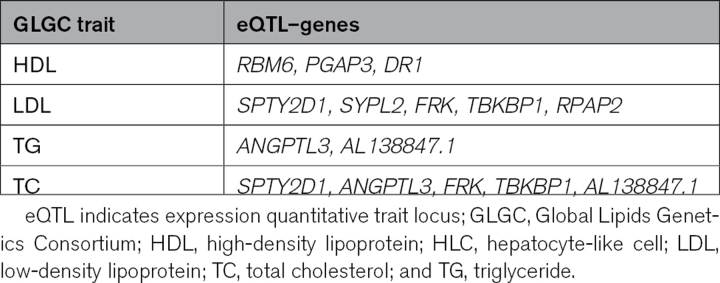
HLC eQTL Genes That Colocalize With GLGC Loci

**Figure 4.**
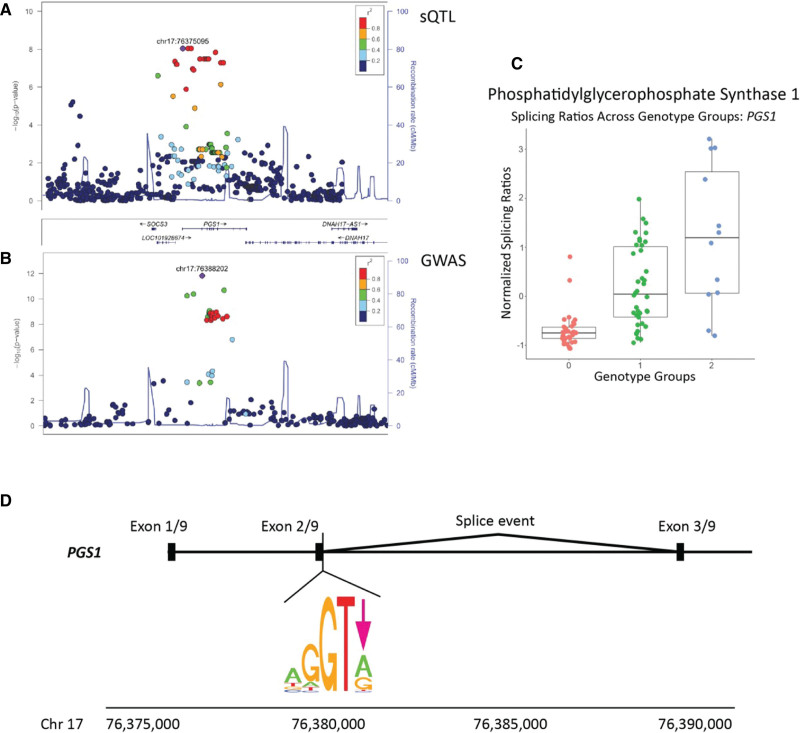
**splicing quantitative trait loci (sQTL) discovery implicates *PGS1* as the effector transcript underlying genome-wide association signal for high-density lipoprotein (HDL) cholesterol levels.** *PGS1* colocalization result: (**A**) sQTL and (**B**) Locuszoom plots for *PGS1* locus, (**C**) boxplots of normalized splicing ratios for sQTL across genotype groups, (**D**) location of credible set single nucleotide polymorphism relative to associated exon-exon junction (indicated by red arrow). Position Weight Matrices used to create the sequence logo obtained from Abril et al.^23^

**Figure 5.**
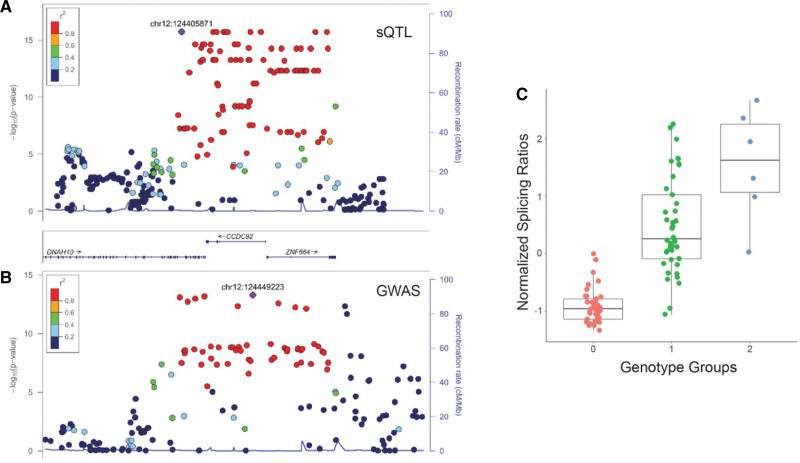
**splicing quantitative trait loci (sQTL) discovery implicates *CCDC92* as the effector transcript underlying genome-wide association signal for high-density lipoprotein (HDL) cholesterol and triglyceride levels.** *CCDC92* colocalization result: (**A**) sQTL and (**B**) HDL locuszoom plots for *CCDC92* locus, (**C**) boxplots of normalized splicing ratios for sQTL across genotype groups for sQTL mapping to *CCDC92*. *CCDC92* indicates coiled-coil domain containing 92.

Of particular interest are the colocalized sQTLs mapping to *CCDC92* and *PGS1* (Figure 4). *PGS1* encodes an enzyme known as phosphatidylglycerophosphate synthase 1, which is involved in the synthesis of the anionic phospholipids phosphatidylglycerol and cardiolipin. SNPs in *PGS1* are associated with changes in triglyceride levels during diabetes treatment.^22^ Interestingly, the second SNP in the credible set for this sQTL falls within the canonical splice site of the splicing event (as demonstrated by the red arrow in Figure 4D).^23^ Furthermore, the differentially spliced intron for *PGS1* is associated with a transcript that is predicted to undergo nonsense-mediated decay (*PGS1-002*), suggesting that the splicing event will have significant impact on levels of functional protein. The *CCDC92-ZNF664* locus has also been implicated in many different metabolic disorders, including type-2 diabetes and coronary heart disease (Figure 5).^24^ The sentinel sQTL SNP at this colocalized locus has been associated with variation in total cholesterol, metabolic syndrome, and waist circumference in previous studies.

## Discussion

In sum, we demonstrate the utility of using iPSC-derived HLCs for the identification of lipid-relevant sQTLs. Our sQTL scans identify thousands of sQTLs in both iPSC and HLC lines, and the sentinel sQTL variants are enriched near canonical splice sites and are located close to their associated splice event, indicating that fine-mapping the causal genes and variants for these sQTLs is feasible. Regarding the degree to which sQTLs are shared across cell types, we find that if a spliced isoform is expressed across both cell types, the sQTL effect is also often shared. Although this finding is in line with previous sQTL analyses, it may be due to the fact that low-expressed splice events are often removed during more stringent filtering processes.

Our colocalization analysis of the HLC sQTLs with GWAS loci from the Global Lipids Genetics Consortium identifies several interesting genes and variants that may underlie blood lipid level variation. Furthermore, the fact that (1) our HLC sQTLs generally colocalize with different GWAS loci than the HLC eQTLs, and (2) the colocalized sQTLs nominate different genes than the eQTLs, indicates that sQTLs likely provide information pertaining to the causal genes and molecular mechanisms underlying complex traits that may not otherwise be captured by eQTL analysis alone.

One limitation of this study is the fact that Leafcutter cannot distinguish between types of sQTL events and may miss variants associated with alternative transcription start sites and alternative polyadenylation. As more differential splicing and sQTL detection methods are published, comparing the results of these various methods may provide a more nuanced assessment of the role of sQTLs in complex disease. Another limitation is that the Gene-Tissue Expression project v6 liver RNA-sequencing data was processed differently than the iPSC and HLC data, which may have resulted in the inability to detect the same sQTLs across cell types at the intron level. The use of primary liver tissue samples to interpret and replicate specific sQTLs we have cataloged here would be an important direction for future work.

In summary, sQTL discovery in iPSC-derived cells may serve as an approach complementary to primary tissue QTL discovery efforts and provide a model system in which to identify and functionally characterize sQTLs relevant for complex trait variation.

## Article Information

### Sources of Funding

Dr Brown acknowledges support from the National Institutes of Health (NIH)–National Heart, Lung, and Blood Institute (HL133218) for the work. Dr Voight is grateful for support from the work from the NIH–National Institute of Diabetes and Digestive Kidney Diseases (DK101478 and DK126194). Dr Gawronski was supported in part from the NIH–National Institute of General Medical Sciences (GM008216) and the American Heart Association (19PRE34450081).

### Disclosures

Dr Voight is an associate editor for *Circulation: Genomics and Precision Medicine*. Dr Pashos was solely affiliated with the University of Pennsylvania at the time of their contribution to this article; their current affiliation is now with Pfizer, Inc. The contents and viewpoints expressed herein and the materials therein reflect the personal opinion of the authors, and cannot be construed as representation of any position or opinion of Pfizer Inc. or any of its subsidiaries. All materials were created at the University of Pennsylvania. The other authors report no conflicts.

### Supplemental Material

Supplemental Methods

Figures S1–S3

References ^25–45^

## Supplementary Material


