# Supplementary material for: Evaluating the Contribution of Cell Type–Specific Alternative Splicing to Variation in Lipid Levels
Source: Circ Genom Precis Med. 2023 May 11;16(3):248–57. doi: 10.1161/CIRCGEN.120.003249 (PMC10284136; doi:10.1161/CIRCGEN.120.003249)
Supplement: Supplementary file 1 [file hcg-16-248-s001.pdf]

SUPPLEMENTAL MATERIAL

Supplemental Methods

iPSC derivation and differentiation into HLCs

As previously described,[15] iPSC cell lines were generated from the peripheral blood mononuclear cells (PBMCs) of 91 human subjects in good health and without cardiovascular disease. In brief, PBMCs were obtained from the subjects' blood, cultured with cytokines, and expanded into erythroblasts. Erythroblasts were then transduced with Sendai viral vectors expressing Oct4, Sox2, Klf-4, and c-Myc. Once transduced, cells were transferred to culture containing mouse embryonic feeder cells. Colonies of iPSC cells were then moved to hESC medium until at least cell passage 12. Once established on the hESC medium, iPSC lines were removed from feeder culture and passaged at least 5 times before use in HLC differentiation. iPSC quality control procedures included mycoplasm testing, quantification of pluripotency markers,[25] and confirmation of Sendai vector loss through RNA-sequencing. HLC differentiation from the iPSCs was conducted using feeder-free differentiation[13]. RNA sequencing was used to confirm the expression of hepatocyte-specific genes in the HLC lines. Albumin, ApoB, and triglycerides were also assayed to confirm HLC quality.

Genotyping and quality control

DNA was extracted from each donor with the QIAsymphony SP system (QIAGEN) and genotyped on the Infinium Human CoreExome-24 BeadChip (Illumina). Variants missing >5% of total genotypes and variants that deviated from Hardy-Weinberg equilibrium were removed. Haplotypes were phased using SHAPEIT2[26] and missing genotypes were imputed to the 1000 genomes phase 3 multi-ethnic panel. Following imputation, the same missingness and Hardy-Weinberg filters were applied to the imputed data.

RNA sequencing data generation

As previously described,[15] RNA was extracted from iPSC and HLC cells using the RNAeasy mini kit (QIAGEN) and assayed with the Agilent RNA 6000 Kit and Bioanalyzer. Libraries were prepared using TruSeq Stranded mRNA Library Prep Kit (Illumina). Sequencing was performed at the University of Pennsylvania's Next Generation Sequencing center using Illumina Hi-Seq 2000/2500 Systems with 100bp/125bp paired-end reads. Target read depth for each sample was 50 million reads. We assessed quality of the raw FastQ files using FastQC[27] and used TrimGalore![28] to trim adaptors. We then aligned trimmed reads that passed FastQC quality control to hg19/GRCh37 reference using the 2-pass mode of STAR aligner.[29] Whole transcriptome RNA-sequencing data was generated for 89 iPSC and 86 HLC samples - we used the data from 83 individuals who had data from both iPSC and HLC lines and that passed differentiation and quality control standards.

Splicing quantification

Alternative splicing events were quantified in both iPSC and HLC samples using Leafcutter 0.2.7 (http://davidaknowles.github.io/leafcutter/index.html, Accessed December, 2017), which uses split-mapped RNA-sequencing reads that span two different exons to identify excised introns. It then identifies groups of excised introns that

share donor/acceptor splice sites. The proportion of reads that map to the individual introns within these intron groups (or "clusters") are then used as quantitative estimates of alternative splicing. The intron clusters used in this analysis were identified using the Leafcutter call: "python leafcutter_cluster.py -j juncfiles.txt -m 50 -o clusters -l 500000."

Differential splicing and expression analysis

We identified introns that are differentially spliced between iPSC and HLC samples using the differential splicing function of Leafcutter.[18] The differential splicing analysis was then run using: "leafcutter_ds.R --num_threads 4 --min_samples_per_group 10 --min_coverage 20 --exon_file=gencode19_exons.txt.gz clusters_perind_numers_noXY.counts.gz SVAconfounderMatrix.txt." The gencode19_exons.txt.gz file is provided on the Leafcutter GitHub, and the SVA confounder matrix was generated using the SVA R package.[30] To control for the fact that our iPSC and HLC samples come from the same donors, we included donor as a covariate in our analyses.

Of the genes that were identified as differentially spliced, we conducted overrepresentation analysis of KEGG Pathway annotations using the R package ClusterProfiler.[31] The background set of genes was comprised of all of the genes that were used for differential splicing analysis (i.e., was "successfully" tested for differential splicing analysis, in that it passed filtering thresholds). To compare iPSC and HLC samples based on patterns of alternative splicing, we conducted principal components analysis on the iPSC/HLC splicing quantifications (normalized, standardized intron ratios) using the R package prcomp.

Using the DESeq2 R package[32], we performed differential expression analysis between the 83 donor-paired iPSC and 83 HLC RNA-Seq samples on all transcripts that had at least 20 samples across all 166 RNA-Seq samples with 6 or more RNA-seq reads mapping. DESeq2 was run with a confounders matrix made up of 23 surrogate variables generated from the quantile normalized, standardized TPMs of the transcripts that met the previously mentioned expression thresholds. To control for the fact that our iPSC and HLC samples come from the same donors, we included donor as a covariate in our analyses. The surrogate variables were generated using the SVA R package.[30] Transcripts were considered differentially expressed in the iPSCs or HLCs if they had a Benjamini and Hochberg adjusted p-value of <0.05 and a $\log_2$-fold-change of 2 or greater.

To compare iPSC and HLC samples based on patterns of gene expression, we also conducted principal components analysis on the iPSC/HLC normalized, standardized TPMs using the R package prcomp.

Finally, we used the ClusterProfiler[31] R package to perform KEGG pathway enrichment analysis on the differentially expressed transcripts in both the HLC samples and the iPSC samples. The pathway enrichment analysis was done with an FDR cutoff of 0.05 and using a background set containing all transcripts that were tested for differential expression.

sQTL identification

Clusters were identified within iPSC and HLC samples using the Leafcutter call: "python leafcutter_cluster.py -j juncfiles.txt -m 50 -o clusters -l 500000." Intron

proportions were standardized across individuals for each intron, and read ratios were quantile-normalized across introns using: “python prepare_phenotype_table.py perind.counts.gz -p1.” We tested for association between variants with MAF>0.05 and within 100kb of a given intron cluster using linear regression as implemented with QTLtools (v2-184, http://fastqtl.sourceforge.net/).[33] To obtain the FDR <5% sQTLs used for the majority of the analyses, we used the “-permute 1000 10000 --grp-best” adaptive permutation command to obtain the most significant variant-intron pair per gene. sQTL scans for both iPSC and HLC samples were also run without permutations for use in qvalue, METASOFT, and colocalization analyses, since permuted results only output p-values for the most highly associated SNP per intron cluster/gene. In each scan we controlled for PEER factors, sex, and four genotype based principal components (calculated on the VCF of all genotypes filtered for variants MAF > 0.05 using EIGENSTRAT’s smartpca – genotypes were first LD pruned with PLINK using R^2=0.8)[34,35]. PEER factors were estimated on the standardized, quantile-normalized splicing phenotypes matrix and were included as covariates in the regression. sQTL scans were run for both iPSC and HLC samples over a range of 1 to 20 PEER factors to determine the number of factors deemed sufficient to correct for potential batch effects for each cell type. Five PEER factors were used in the HLC sQTL scan, while six PEER factors were used in the iPSC sQTL analysis. QTLtool’s adaptive permutations scan outputs p-values adjusted for the number of introns tested per gene. We then further corrected these p-values across genes using the Benjamini and Hochberg method to obtain a global false discovery rate[36].

For the sQTL scan conducted in the GTEx v6 liver samples (n=96), a similar procedure was followed in order to obtain the nominal sQTLs used for the replication analysis. We used regression as implemented by FastQTL[37] to obtain the nominal p-values used for replication qvalue analyses. Due to differences in data processing and genetic ancestry of the samples, 3 genotype PCs and 5 PEER factors were included as covariates.

*Acquisition of genomic annotation*s

Splice site windows were obtained by taking Gencode v19 exon annotations and setting intervals 3bp into an exon and 8bp beyond an exon boundary to encompass the range of base pairs that encompass canonical binding sites for splicing machinery. Genomic regions were ascertained from Gencode v19 annotations using the GenomicFeatures R package.[38] We retained known protein coding transcripts, and then obtained annotations for introns, 5’ UTR, 3’ UTR, coding sequence, and promoters using the intronsByTranscript, fiveUTRsByTranscript, threeUTRsByTranscript, cdsBy, and promoters functions, respectively. RNA binding protein peaks were downloaded from eCLIP files from ENCODE.[39] Using the data from the same eCLIP paper that provided the ENCODE RBP files, we identified 41 RBPs implicated in either splicing regulation of 3’ processing. For each RBP, we took the intersection of each of the replicate peak files, then combined all intersected files to get one BED file of “RBP peaks.”

Distance to 5’ and 3’ ends of genes and location within introns

sQTL variants were identified as being upstream, downstream, or within the excised intron with which they were associated. We then plot the distance of the variant to the 5’ or 3’ end of the intron cluster. If the sQTL variant fell in between the exons within the excised intron, intron length was divided by 10 and the sQTL was assigned to one of these deciles.

Replication analysis in primary liver tissue

We used the R package qvalue[17] to calculate the π_0 values for the P-value distribution of iPSC and HLC sQTL SNP-gene pairs in GTEx v6 liver data. For each of the SNP-gene pairs in the FDR < 5% iPSC and HLC sQTL scan, we pulled the nominal p-values for these same pairs out of the GTEx v6 liver sQTL scan to obtain an estimate of the replication rate.

Cell-type specificity analyses

To evaluate the degree to which sQTLs are shared between cell-types, we used METASOFT[40] to calculate the posterior probabilities (m-values) that an sQTL discovered in iPSC samples is also an sQTL in HLC samples, and vice-versa. An sQTL was considered associated with both cell types if the m-value was ≥0.9 for both cell-types. An sQTL was considered HLC-specific if the m-value was ≥0.9 in HLCs and <0.1 in iPSCs. Similarly, sQTLs were considered iPSC-specific if the m-value was ≥0.9 in iPSCs and <0.1 in HLCs.[41] To facilitate comparisons between sQTLs and eQTLs, we conducted METASOFT analysis on the top one thousand sQTLs from our iPSC and HLC permutation scans. The same procedure was used for our iPSC and HLC eQTLs.

eQTL identification

Aligned RNA-seq reads were assigned to Gencode v19 genomic annotations using the R package featureCounts.[42] Transcripts were filtered for HLC and iPSC samples separately and were kept for analysis if at least 10 samples had 6 or more mapped RNA-seq reads. Raw featureCounts transcript counts were converted to TPMs. For each sample, the distribution of TPMs were normalized to the empirical average quantiles across samples using the R function normalizeQuantiles, and then, for each gene, for each cell type, the distribution of TPMs was transformed to the quantiles of the standard normal distribution, using the R function qqnorm. We tested for association between bi-allelic variants with MAF>0.05 and within 1Mb of a given transcript (beginning or end of transcript) using linear regression as implemented with QTLtools (v2-184, http://fastqtl.sourceforge.net/). To obtain the FDR <5% eQTLs used for the majority of the analyses, we used the “--permute 1000 10000” adaptive permutation command to get the most significant variant-gene pair. eQTL scans for both iPSC and HLC samples were also run without permutations for use in METASOFT and colocalization analyses, since permuted results only output p-values for the most highly associated SNP per gene.

As with the sQTL analysis, in each eQTL scan we controlled for PEER factors[43], gender, and four genotype based principal components (calculated using EIGENSTRAT’s smartpca, and first LD pruned R^2=0.8 with PLINK).[34,35] PEER factors were estimated using on the standardized, quantile-normalized TPMs calculated from the featureCounts output and were included as covariates in the regression. eQTL

scans were run for both iPSC and HLC samples over a range of 1-30 PEER factors to determine the number of factors that maximized the numbers of eQTLs discovered for each cell type. 20 PEER factors were used in the HLC eQTL scan, while 16 PEER factors were used in the iPSC eQTL scan. We used the same adaptive permutation QTLtools function as for our sQTL scans to output p-values adjusted for the number of variants tested per gene. We then further corrected these permuted p-values across genes using the Benjamini and Hochberg method to obtain a global FDR.[36]

Credible Set Calculations

To calculate 95% credible sets for each of the iPSC/HLC FDR < 5% sQTLs, we used the process described in Maller et al. 2012 to identify sets of variants with a 95% probability of containing the causal variant.[44]

Colocalization analyses

Using the default parameters for the R package coloc[20] we conducted colocalization analysis between each of the GWAS traits from the Global Lipids Genetics Consortium[1] (HDL-C, LDL-C, TG, and TC) and the FDR <5% eQTL-gene and sQTL-gene pairs from our HLC samples. For each GLGC trait, we ranked genome-wide significant variants by p-value and chose the most significant variants within 100kb windows of each other. If any of the FDR <5% eQTL/sQTL variants fell within 100kb of these GLGC variants, we conducted colocalization analysis on all variants within 100kb of the GWAS variants, using the p-values obtained from our nominal sQTL/eQTL scans. We used the MAF estimated from the GLGC.

Enrichment analysis using GoShifter.

GoShifter[45] was used to determine the degree to which iPSC and HLC sQTLs (FDR <5%) were enriched in the annotations described above. We used LD files calculated with PLINK[35] on our FDR <5% sQTLs to get the groups of SNPs in LD of R^2>0.8 required for running GoShifter, which creates “circularized” loci and shifts annotations within these regions to provide a null distribution with which to compare the observed results. Odds ratios were calculated using the fisher.test function in R.

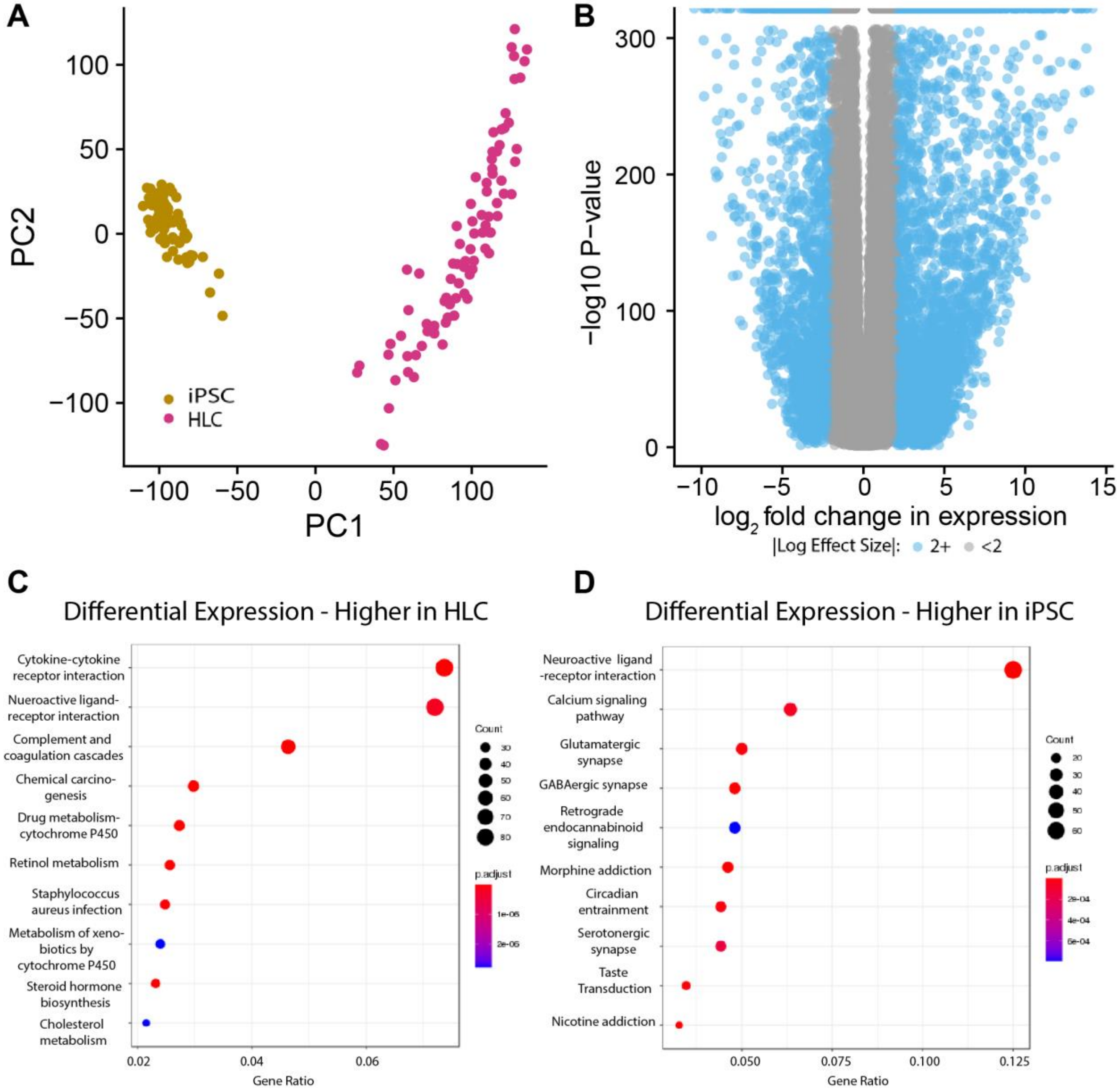

Supplementary Figure I. Genes that are differentially expressed between iPSC and HLC samples (FDR <5%): a) PCA of normalized gene expression (TPMs), b) Volcano plot of differentially expressed genes (FDR <5%), c) KEGG pathway enrichment analysis for genes that are differentially expressed more highly in HLC relative to iPSC, d) KEGG pathway enrichment analysis for genes that are differentially expressed less highly in HLC relative to iPSC. The top third of enriched pathways are plotted above.

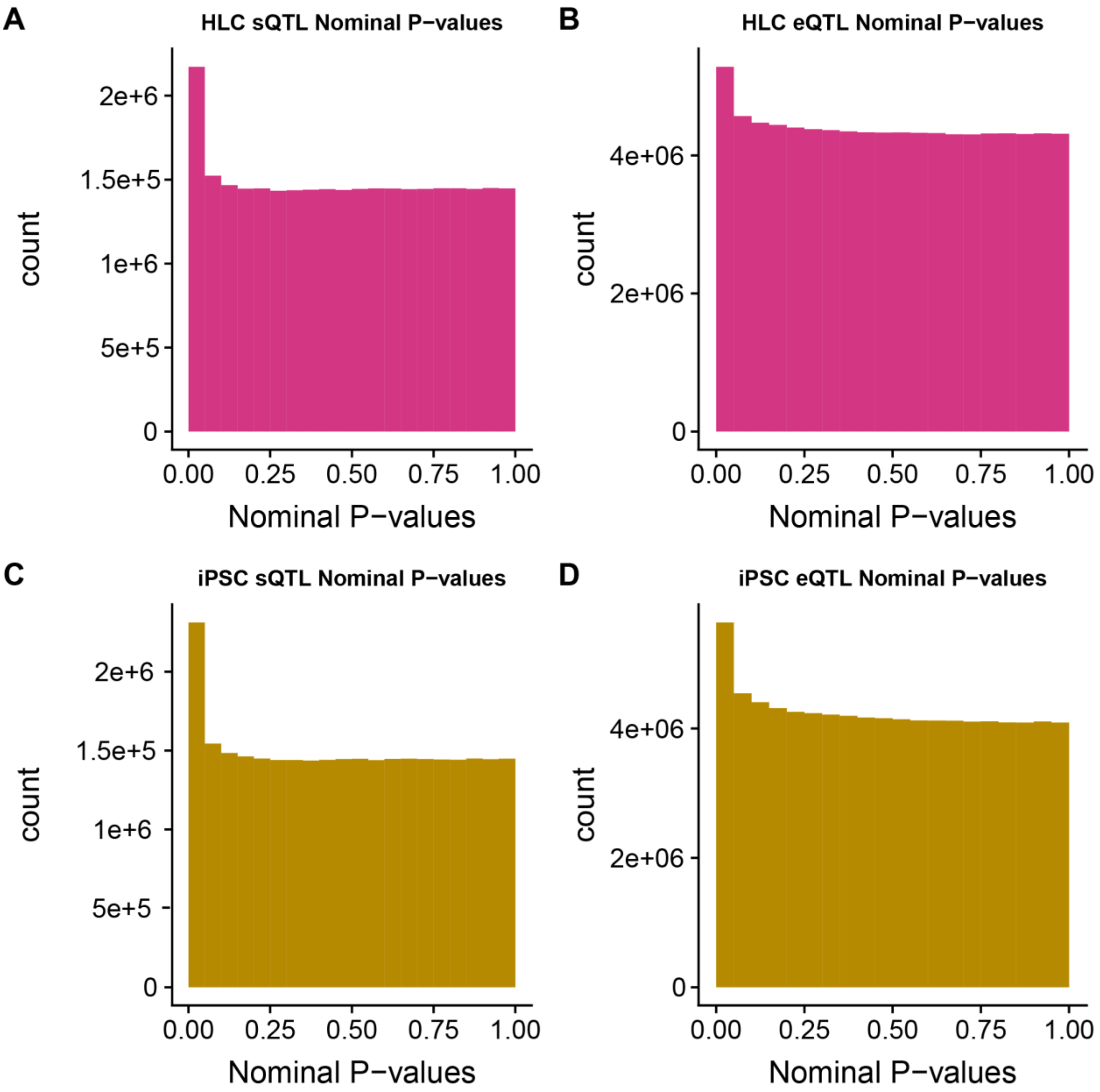

Supplementary Figure II. P-value histograms for sQTL and eQTL scans.

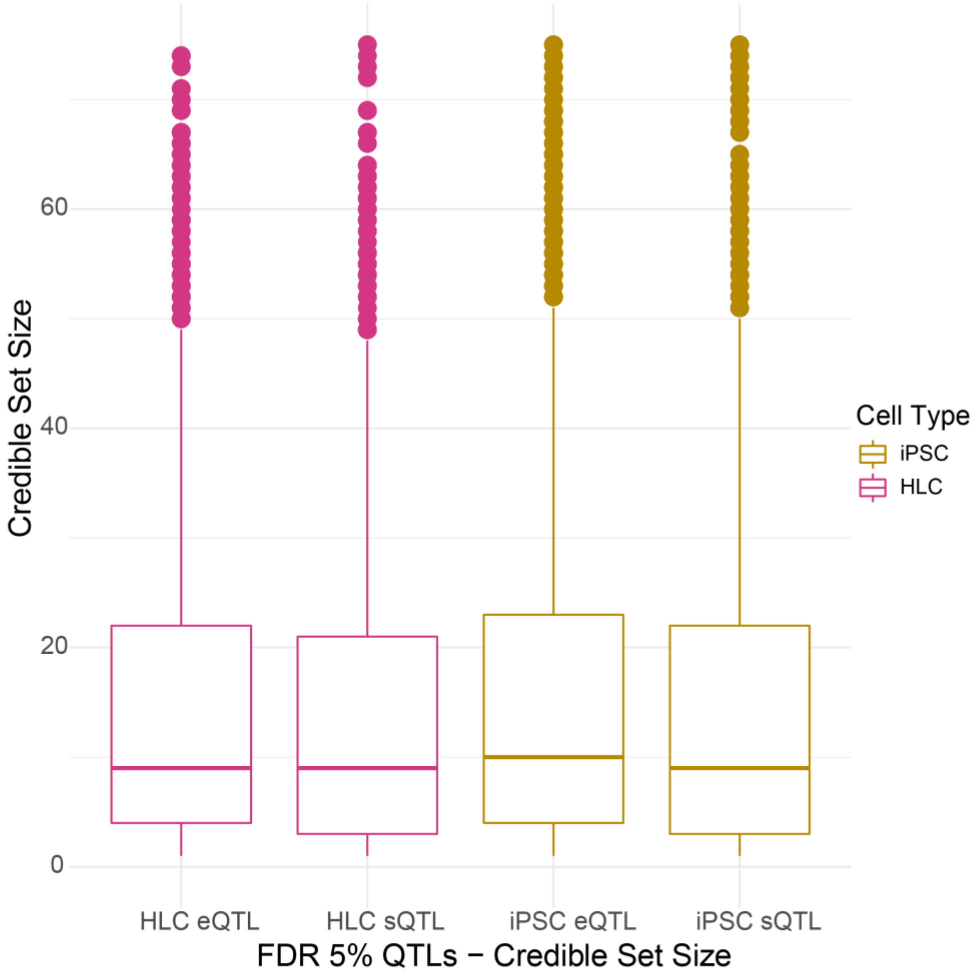

Supplementary Figure III. Credible sets for iPSC, HLC sQTLs and eQTLs. Boxplot whiskers calculated as 1.5 * interquartile range.
